# Obesity‐induced reduction of adipose eosinophils is reversed with low‐calorie dietary intervention

**DOI:** 10.14814/phy2.13919

**Published:** 2018-11-22

**Authors:** William Reid Bolus, Arion J. Kennedy, Alyssa H. Hasty

**Affiliations:** ^1^ Department of Molecular Physiology and Biophysics Vanderbilt University School of Medicine Nashville Tennessee; ^2^ VA Tennessee Valley Healthcare System Nashville Tennessee

**Keywords:** Adipose tissue, eosinophils, inflammation, macrophages, obesity, weight loss

## Abstract

While many studies have characterized the inflammatory disposition of adipose tissue (AT) during obesity, far fewer have dissected how such inflammation resolves during the process of physiological weight loss. In addition, new immune cells, such as the eosinophil, have been discovered as part of the AT immune cell repertoire. We have therefore characterized how AT eosinophils, associated eosinophilic inflammation, and remodeling processes, fluctuate during a dietary intervention in obese mice. Similar to previous reports, we found that obesity induced by high‐fat diet feeding reduced the AT eosinophil content. However, upon switching obese mice to a low fat diet, AT eosinophils were restored to lean levels as mice reached the body weight of controls. The rise in AT eosinophils during dietary weight loss was accompanied by reduced macrophage content and inflammatory expression, upregulated tissue remodeling factors, and a more uniformly distributed AT vascular network. Additionally, we show that eosinophils of another metabolically relevant tissue, the liver, did not oscillate with either dietary weight gain or weight loss. This study shows that eosinophil content is differentially regulated among tissues during the onset and resolution of obesity. Furthermore, AT eosinophils correlated with AT remodeling processes during weight loss and thus may play a role in reestablishing AT homeostasis.

## Introduction

Obesity has been one of the fastest growing factors negatively influencing human health over the last several decades (Flegal et al. 2012; Ogden et al. 2015; CDC, 2016). In particular, obesity increases the risk for type 2 diabetes, insulin resistance, cardiovascular disease, certain cancers, infertility, asthma, and many other diseases (Shoelson et al. 2007; Khalid and Holguin 2018; Stone et al. 2018). Indeed, a report of 57 prospective analyses comprised of nearly a million adults, showed that obesity can decrease a person's life span by up to 10 years (Prospective Studies et al. 2009). Therefore, a better understanding of the processes regulating obesity, both during weight gain and weight loss, is vital to combating the obesity epidemic.

It may come as no surprise that adipose tissue (AT) undergoes massive expansion during obesity, sometimes accounting for over 50% of a person's body mass. AT can expand in a healthy way to a degree, but a limit is reached, and further expansion leads to AT dysfunction marked by hypoxia, adipocyte cell death, fibrosis, and a complete overhaul of the immune cell populations (Sun et al. 2011; Hill et al. 2014). Indeed, healthy lean AT contains an assortment of immune cells including, but not limited to, anti‐inflammatory M2‐like macrophages, Th2 CD4^+^ T cells, regulatory T cells, and eosinophils (Wu et al. 2011; Winer and Winer 2012). In contrast, immune cells of obese AT consist of the proinflammatory counterparts (i.e., M1‐like macrophages) and/or inversed quantities of anti‐inflammatory cells (i.e., reduced eosinophils).

Eosinophils, and other Th2‐like innate immune cells, have risen to the forefront of recent AT research. Many studies indicate eosinophils and innate lymphoid type 2 cells are key players in maintaining AT homeostasis, but these studies are not without their caveats (Bolus and Hasty 2018; Knights et al. 2018). While high levels of systemically elevated eosinophils have been shown to protect against the effects of high‐fat diet (HFD)‐induced obesity (Wu et al. 2011; Molofsky et al. 2013; Hussaarts et al. 2015), restoring AT eosinophils to physiological levels has not recapitulated such beneficial effects (Bolus et al. 2017). These mixed results demonstrate the need for continued research to determine the exact conditions that allow eosinophils to regulate AT homeostasis (i.e., which circumstances are permissive to eosinophil‐mediated improvements in metabolic health, and which are not). While many studies continue elucidating how eosinophils behave during weight gain, no studies to date have examined eosinophils of obese mice during dietary weight loss. A single study has shown that calorically restricting lean mice, which already have sufficient levels of AT eosinophils, can further increase eosinophil numbers and subsequently demonstrate improved cold tolerance (Fabbiano et al. 2016). However, whether the reduced AT eosinophil numbers during obesity return to lean levels after weight loss has not been examined. Furthermore, the effect of weight gain and loss on eosinophils in other metabolic tissues such as the liver has not been reported.

In the current study, we tracked eosinophil populations in both AT and liver throughout a detailed time course of diet‐induced weight loss in obese mice. We show, for the first time, that the reduced AT eosinophils of obese mice are restored to physiological lean levels by dietary intervention, occurring alongside macrophage inflammatory resolution. Furthermore, we report the novel finding that liver eosinophil numbers are not compromised during dietary weight gain or subsequently altered during dietary weight loss, despite fluctuations in macrophage (Kupffer cell) populations.

## Materials and Methods

### Animals

C57BL/6J male wild type mice purchased from the Jackson Laboratory (Bar Harbor, Maine) at 7 weeks of age were acclimated for 1 week in the animal facility at Vanderbilt University. At 8 weeks of age, mice were given ad libitum access to either low fat diet (LFD) with 10% kcal from fat (D12450B), or HFD with 60% kcal from fat (D12492) (Research Diets, New Brunswick, NJ) for 12 weeks to induce dietary weight gain; diets are matched for micronutrient content. After 12 weeks of weight gain, mice fed a HFD were switched to LFD to induce weight loss for up to 6 weeks; mice previously on LFD remained on LFD. Mice were weighed weekly for the duration of the study. Mice were examined at 0, 3, 7, 14, 21, and 42 days postdiet switch. HFD and LFD groups had 36 and 18 mice/group, respectively. The HFD group decreased by six mice beginning at day 0 of diet switch, and at each subsequent day noted. The LFD control group had eight mice/group throughout weight loss of the HFD group. At each endpoint, circulating blood was removed by perfusing the left ventricle of the heart with ~10–15 mL PBS. Individual tissues were removed, weighed, and either snap‐frozen in liquid nitrogen, fixed in 1% PFA, or processed to isolate immune cells, in preparation for later analysis. Vanderbilt's Institutional Animal Care and Use Committee approved all animal procedures prior to their implementation.

### Immune cell isolation and flow cytometry

#### AT stromal vascular fraction cell isolation

AT was excised and minced into a slurry in a 1% FBS PBS solution. Minced AT was digested for 40 min at 37°C at 200 rotation (MaxQ4450, Thermo Fisher Scientific, Middletown, VA) with 2 mg/mL type II collagenase (Millipore‐Sigma, St. Louis, MO). PBS with 1% FBS solution was added to dilute the collagenase solution fourfold. Solution was homogenized by briefly vortexing and passed through a 100 *μ*m filter. After centrifugation, the cell pellet was resuspended in ACK lysis buffer to remove red blood cells. Reaction was neutralized by dilution with 1% FBS PBS, centrifuged, and decanted; cell pellet was resuspended and used for further analysis.

#### Liver nonparenchymal cell isolation

Liver was excised and minced into fine pieces in 1 mg/mL type II collagenase (Millipore‐Sigma) in 3% FBS PBS solution. Minced liver was incubated for 30 min at 37°C at 200 rpm rotation (MaxQ4450). Cell suspension was filtered through a 100 *μ*m filter, with added manual force to ensure tissue was thoroughly filtered, and subsequently centrifuged for 3 min at 15*g* (Sorvall ST 40R, Thermo Fisher Scientific). The supernatant was collected and centrifuged for 10 min at 350*g*. The pellet was resuspended in 40% Percoll and overlaid on top of 60% Percoll. The Percoll gradient was centrifuged at 625*g* for 20 min. The two middle layers of the Percoll gradient were collected in 3% FBS in RPMI and centrifuged for 10 min at 350*g*. Supernatant was decanted; cell pellet was resuspended and used for further analysis.

#### Flow cytometric analysis

Isolated immune cells were incubated for ≥5 min with Fc Block (BD Biosciences, San Jose, CA) on ice. Cells were stained for 30 min at 4°C, concealed from light, with a combination of fluorophore‐conjugated antibodies: F4/80: APC (eBiosciences, Waltman, MA), CD11b: FITC/APC‐Cy7/APC (BD Biosciences), SiglecF: PE/BV450 (BD Biosciences), CD45: PE‐Cy7(BD), APC‐Cy7/BV605 (Biolegend), and CD19: APC‐Cy7 (BD Biosciences). Cells were washed several times, counting beads added, and stained with viability dye, (1 *μ*g/mL 4’,6‐diamidino‐2‐phenylindole [DAPI] or propidium iodide [PI]), just before flow cytometric analysis. Cells were analyzed on a 4‐laser BD LSR Fortessa (BD Biosciences) in the Vanderbilt Flow Cytometry Shared Resource (FCSR). Eosinophils were gated as DAPI‐ (live), CD45+, F4/80^lo^, CD11b^lo^, SiglecF+ cells. Macrophages were gated as DAPI‐ (live), CD45+, F4/80^hi^, CD11b^hi^, SiglecF‐ cells. Results were analyzed using FlowJo software.

### RNA isolation, cDNA synthesis, and real‐time RT‐PCR

The Qiagen RNeasy Mini Kit was used to isolate RNA according to the manufacturer's instructions, after tissues were initially homogenized in TRIzol (BD Biosciences, Hercules, CA). Purified RNA was reverse transcribed by iScript RT (Bio‐Rad) into cDNA. Differences in relative gene expression were quantified using FAM‐conjugated TaqMan Gene Expression Assay (Thermo Fisher, Middletown, VA, USA). The following genes were examined by RT‐PCR: *Siglecf* (Mm00523987_m1; Thermo Fisher), *Prg2* (Mm0043905_m1), *Ccr3* (Mm00515543_s1), *Emr1* (Mm00802529_m1), *Tnfa* (Mm00443258_m1), *Itgax* (Mm00498698_m1), *Arg1* Mm01190441_g1), *Mmp9* (Mm00442991_m1), *Vegfa* (Mm01281449_m1), *Fgf2* (Mm00433287_m1), and *Gapdh* (Mm99999915_g1). Data were normalized to the housekeeping gene, *Gapdh*, and analyzed by the Pfaffl method (Pfaffl 2001).

### Vascular imaging by CD31 immunofluorescence

Small pieces of whole AT were fixed in 1% PFA for 1 h with agitation. AT was washed several times in PBS with agitation and stored in PBS at 4°C until further processing. AT was cut into ~3 mm^3^ pieces and incubated overnight at 4°C with slow agitation in anti‐mouse CD31 antibody. The next day, samples were washed in PBS and incubated for 1 h at RT with anti‐rat FITC secondary antibody. Samples were washed a final time, incubated with 1 *μ*g/mL DAPI (BD Biosciences) for 3 min at RT with agitation, and mounted in a 35 mm petri dish with #1.0 coverglass bottom (MatTek, Ashland, MA). Samples were imaged on an Olympus FV‐1000 confocal inverted microscope available through the Vanderbilt Cell Imaging Shared Resource (CISR). Each image stack spanned ~30 *μ*m (1 image/1 *μ*m) and was then compiled into a single 3D rendering using Imaris software.

### Statistics

All statistical graphs and analyses were performed in GraphPad Prism 7.0 software. Statistical tests include: student's *t‐*test and one‐way ANOVA with a Holm‐Sidak post hoc test for multiple comparisons. Outliers were removed by the ROUT method, with Q = 5%. Significance was defined by a *P* ≤ 0.05. The particular statistical test for each data set is listed in the corresponding Figure legend.

## Results

### Diet‐induced weight loss study design

**Figure 1:**
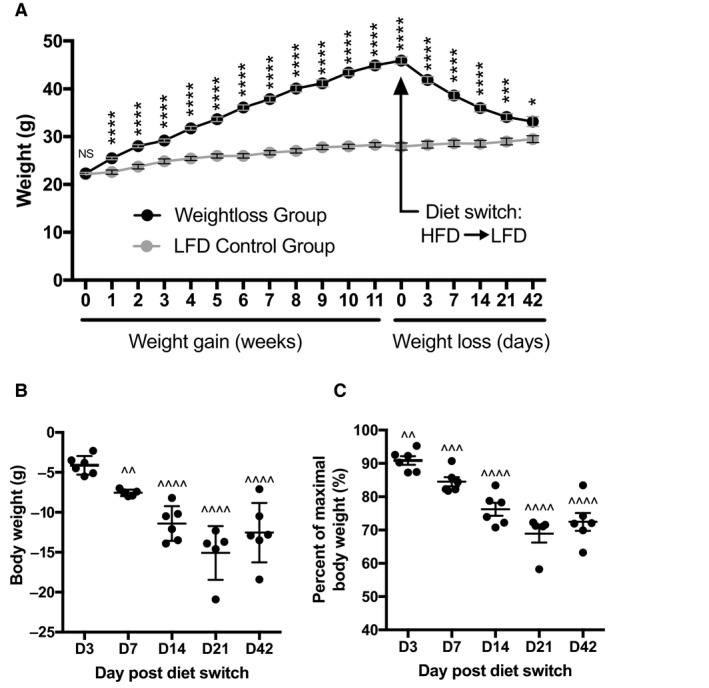
Weight loss study design and associated body weight parameters. (A) Body weight (g) of mice placed on high‐fat diet (HFD) (black circles) or control low fat diet (LFD) (gray circles) for 12 weeks, at which point HFD‐fed mice were switched to LFD (day 0); weights were also recorded at day 3, 7, 14, 21, & 42 postdiet switch. (B) Change in grams of body weight at each time point during low‐calorie dietary intervention compared to respective D0 weight. (C) The percent body weight at each time point postdietary intervention of maximal body weight gained immediately prior to LFD diet switch. Data are presented as mean ± SEM. Prior to weight loss, HFD and LFD groups had 36 and 18 mice/group, respectively. The HFD group decreased by six mice beginning at day 0 of diet switch, and at each subsequent day noted. The LFD control group had eight mice/group throughout weight loss of the HFD group. *P < 0.05; ***P < 0.001; ****P < 0.0001 compared to LFD control group. ^^*P* < 0.01; ^^^*P* < 0.001; ^^^^*P* < 0.0001 compared to respective HFD D0. *T*‐tests were used for all analysis.

C57BL/6J mice were placed on a 60% HFD to induce weight gain or a 10% LFD control for 12 weeks (Fig. 1A). Weight gain was evident in HFD‐fed mice compared to LFD controls within 1 week of diet and continued to rise steadily throughout the 12‐week period. At 12 weeks of diet, HFD‐fed mice were switched to LFD to induce weight loss while LFD controls remained on LFD. Dietary‐induced weight loss was observed as soon as 3 days postdiet switch and continued for 42 days until nearly reaching LFD controls. The change in body weight post HFD to LFD switch was quantified per gram at days 3, 7, 14, 21, and 42 (Fig. 1B). Weight loss data are also represented postswitch to LFD as a percent of the maximal body weight gained at the peak of HFD feeding (Fig. 1C).

### Effect of dietary weight loss on individual tissue mass

**Figure 2:**
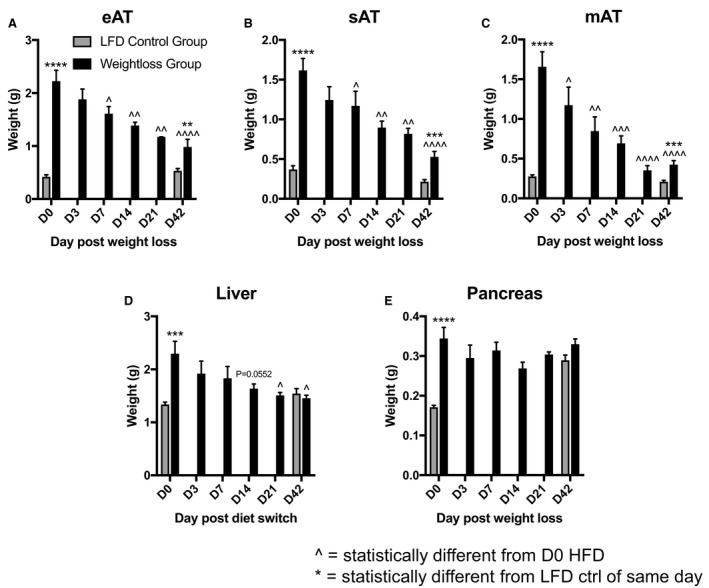
Mass of tissues during low‐calorie dietary intervention. Total weight (g) of each tissue throughout diet‐induced weight loss (HFD→LFD) compared to LFD control, including (A) epididymal AT (eAT), (B) subcutaneous AT (sAT), (C) mesenteric AT (mAT), (D) liver, and (E) pancreas. Data are presented as mean ± SEM. LFD control = 8–9 mice/group; HFD = 4–6 mice/group. ***P < 0.001; ****P < 0.0001 compared to LFD control group. ^*P* < 0.05; ^^*P* < 0.01; ^^^*P* < 0.001; ^^^^*P* < 0.0001 compared to HFD D0. *T*‐tests were used to compare HFD to LFD groups. One‐way ANOVA was used to compare HFD D0 to all other HFD time points.

Tissue weights of epididymal adipose tissue (eAT) (Fig. 2A), inguinal subcutaneous AT (sAT) (Fig. 2B), mesenteric AT [mAT] (Fig. 2C), liver (Fig. 2D), and pancreas (Fig. 2E) were recorded throughout dietary weight loss following a switch from HFD to LFD. At the peak of 12‐week HFD feeding, mass of all tissues examined was higher in HFD‐fed mice compared to LFD controls (Fig. 2A–E). Upon switch from HFD to LFD, both eAT and sAT reached statistically lower weights by D7 compared to HFD D0 (Fig. 2A–B), while mAT reached a lower weight sooner at D3 (Fig. 2C). In contrast, liver did not reach a statistically lower weight until D21 of dietary intervention, though it nearly decreased earlier at D14 with a *P*‐value of 0.0552 (Fig. 2D). Pancreas weight among the HFD groups remained unchanged throughout (Fig. 2E), though LFD control pancreas weight increased to that of dieted switch pancreas weight by the study's end (Fig. 2E).

### Restoration of modified adipose eosinophil and macrophage levels with dietary weight loss

**Figure 3:**
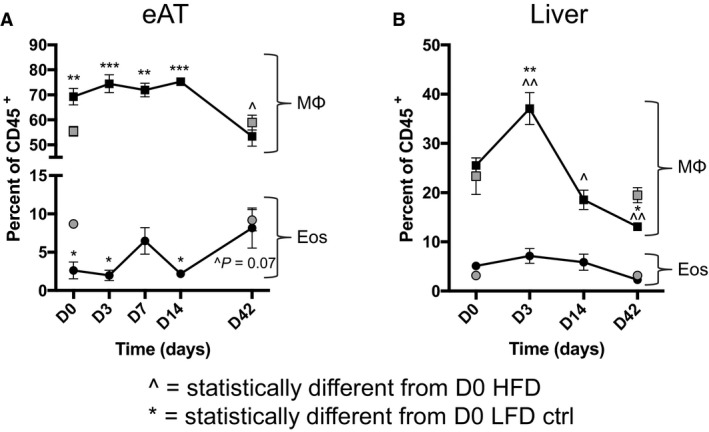
Variation in eosinophil and macrophage content during low‐calorie dietary intervention. Fluctuation in the percent of eosinophil and macrophage populations from mice undergoing dietary weight loss (black circles) compared to LFD controls (gray circles), in (A) eAT and (B) liver. Data are presented as mean ± SEM of 5–6 mice/group. *P < 0.05; **P < 0.01; ***P < 0.001 compared to D0 LFD control group. ^*P* < 0.05; ^^*P* < 0.01 compared to D0 HFD group. One‐way ANOVA was used to compare HFD D0 to all other HFD time points, as well as to compare LFD D0 to all HFD time points.

The percent of eAT macrophages was higher in HFD‐fed obese mice compared to LFD‐fed controls at 12 weeks of weight gain (Fig. 3A). By 42 days of diet switch from HFD to LFD, eAT macrophage numbers were restored to LFD control levels. In contrast, the percent of eAT eosinophils was lower in HFD‐fed mice compared to lean controls, as has been reported (Fig. 3A; (Wu et al. 2011; Molofsky et al. 2013; Hussaarts et al. 2015; Bolus et al. 2017, 2015; Amano et al. 2014; Molofsky et al. 2015; Ding et al. 2016; van den Berg et al. 2017). Diet‐induced weight loss returned the percentage of eAT eosinophils to those found in LFD control mice by 42 days of LFD intervention, with a temporary surge in eosinophils at day 7 (Fig. 3A). While there was an increase in liver macrophages (Kupffer cells) at day 3 of diet‐induced weight loss, the increase was normalized by the final 42‐day time point (Fig. 3B). In contrast to eAT, liver eosinophils were not lower in obese HFD‐fed mice compared to LFD controls and neither group presented any fluctuations in liver eosinophils during or at the conclusion of diet‐induced weight loss (Fig. 3B).

### Alterations in inflammatory and tissue remodeling factors over time during dietary weight loss

**Figure 4:**
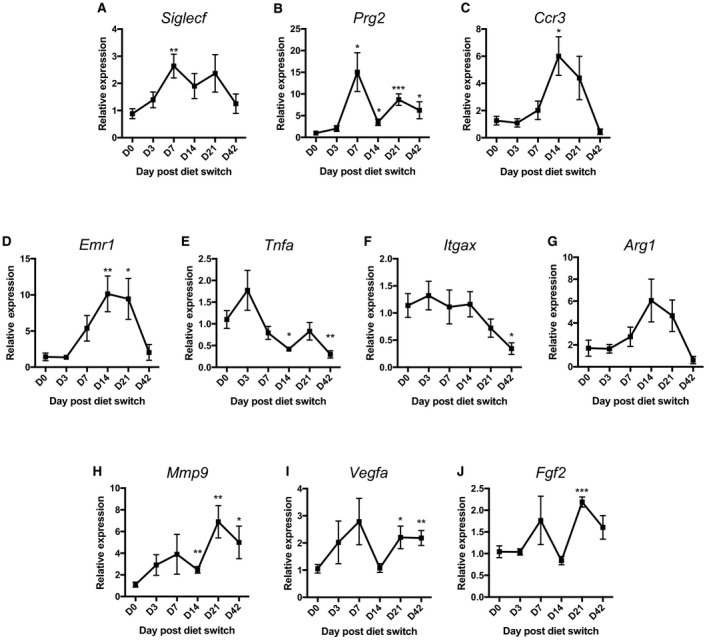
Inflammatory and tissue remodeling gene expression profile of adipose tissue during diet‐induced weight loss. (A–C) Eosinophil‐associated genes during dietary weight loss, including (A) *Siglecf*, (B) *Prg2*, (C) *Ccr3*. Macrophage marker (D) *Emr1*, proinflammatory macrophage markers (E) *Tnfa* and (F) *Itgax* and anti‐inflammatory macrophage marker (G) *Arg1*. Tissue remodeling factors (H) *Mmp9*, (I) *Vegfa*, and (J) *Fgf2*. Data are presented as mean ± SEM of 4–6 mice/group. *P < 0.05; **P < 0.01; ***P < 0.001 compared to D0 time point. One‐way ANOVA was used to compare HFD D0 to all other HFD time points.

Due to the fluctuations in eAT immune cell numbers observed by flow cytometry during dietary weight loss, inflammatory gene expression was examined in the HFD‐fed group of mice upon exposure to LFD (Fig. 4). Eosinophil markers, *Siglecf*,* Prg2*, and *Ccr3* were assessed across a number of time points during weight loss (Fig. 4A–C). *Siglecf* and *Prg2* exhibited increased peaks of expression at day 7 when the increase in eAT eosinophils was also observed by flow cytometry, and *Prg2* remained elevated for the remaining period of weight loss (Fig. 4A and B). Expression of *Ccr3* peaked at day 14 but returned to baseline by the end of the weight loss period (Fig. 4C). The common macrophage marker *Emr1* (more recently referred to as *Adgre1*) increased in expression by day 14 and day 21 but returned to baseline by day 42 of dietary intervention. Upon induction of weight loss, the inflammatory M1‐like marker *Tnfa* was reduced by day 14 (Fig. 4E), and along with M1‐like marker *Itgax*, was both decreased by day 42 (Fig. 4E and F). Though not statistically significant, expression of M2‐like marker *Arg1* closely trended with the same expression pattern of *Emr1* over time (Fig. 4G). Because of the dependence of AT homeostasis on changes in inflammation, we also evaluated the tissue remodeling factors *Mmp9*,* Vegfa*,* Fgf2* (Fig. 4H–J). The matrix metalloproteinase, *Mmp9*, was the first to increase, at day 14 of weight loss (Fig. 4H). All three tissue remodeling factors (*Mmp9*,* Vegfa*,* Fgf2*) were increased by day 21 of weight loss (Fig. 4H–J, and *Mmp9* & *Vegfa* remained elevated at the final weight loss time point (Fig. 4H and I).

### Architectural transformation of adipose vasculature during dietary weight loss

**Figure 5:**
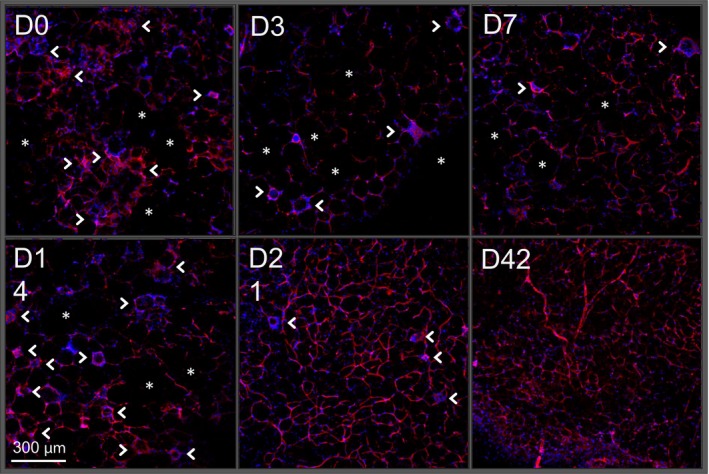
Architectural transformation of adipose vasculature during dietary weight loss. Visualization of adipose vascular network by CD31 immunohistochemistry (red) beginning at the height of weight gain (D0) and continuing through the course of diet‐induced weight loss (D3‐D42). Nuclei are stained with DAPI (blue). Asterisks = areas devoid of vasculature. Arrowheads = crown‐like structures. Images are representative of 4–5 mice/group.

Given the fluctuations in tissue remodeling factors during dietary weight loss, we examined the eAT vascular network, starting at the height of HFD‐induced obesity and subsequently throughout dietary weight loss for 42 days (Fig. 5). Before switching from HFD to LFD, the AT vasculature of obese mice was highly variable, exhibiting regions of dense and irregular vascular development while pockets devoid of a vascular bed remained common (indicated by asterisks). There was also marked high cellular infiltrate, most likely macrophages, as this is commonly observed in eAT of obese mice. As early as 3 days of diet‐induced weight loss, the initial loss of the dense irregular vascular network was observed. By day 7, a slight but somewhat uniform increase in vasculature was present. Day 14 showed no apparent change in the vascular network, but there was an increase in cellular density in discreet pockets, often referred to as crown‐like structures, and typically heavily populated by macrophages (indicated by arrowheads). By day 21, when gene expression of all three tissue remodeling factors was increased (Fig. 4H–J), there was an evenly distributed network of vasculature throughout the eAT, which remained intact, as seen on day 42 of weight loss.

## Discussion

In this study, we show that obese AT eosinophil numbers can be restored over time when mice lose weight due to a dietary intervention, that is, switching from HFD to LFD. The increase in AT eosinophils occurs concomitantly with a decrease in inflammatory macrophages. It is unclear if during weight loss, the AT eosinophils regulated macrophage levels, or vice‐versa, or if each is independently regulated, as each cell type has been shown capable of influencing the other in disparate settings (Voehringer et al. 2007; Wu et al. 2011).

In addition to examining AT eosinophil numbers, we also looked at several eosinophil‐associated genes. The rise in AT eosinophils during weight loss was confirmed by gene expression of eosinophil granule‐specific marker *Prg2* (major basic protein), but not *Siglecf* or *Ccr3*. *Prg2* is one of four primary constituents of eosinophil granules and would presumably increase positively with eosinophil numbers or activation. *Ccr3* is a G protein‐coupled receptor primarily expressed on eosinophils and regulates their differentiation and recruitment most notably in response to eotaxins (Daugherty et al. 1996; Lamkhioued et al. 2003). *Ccr3* expression was most significantly upregulated prior to the increase in eosinophil numbers quantified by flow cytometry. A subsequent decline in AT *Ccr3* expression at the end of the weight loss period may be in response to eosinophils reaching sufficient homeostatic levels. *Siglecf* is known to regulate eosinophil apoptosis and accumulation, with downregulation associated with greater eosinophil numbers (Mao et al. 2013); therefore, the low *Siglecf* levels observed at the end of weight loss may reflect the preservation of eosinophils seen by flow cytometric analysis.

The restoration of AT eosinophils during weight loss was accompanied by increased expression of remodeling factors *Mmp9*,* Vegfa*, and *Fgf2*, all of which can be expressed by eosinophils (Horiuchi and Weller 1997; Levi‐Schaffer et al. 1999; Hoshino et al. 2001). Along with increased remodeling factors, there was a distinct change in the AT vasculature during weight loss. At the height of obesity, the vascular network was not evenly distributed. There is an initial observed decrease in the vascular bedding by D3 that appears to be rebuilt and developed as weight loss progresses. Between days 14 and 21 postdiet‐induced weight loss, the greatest expansion of the vascular network can be observed. It is not clear from this experimental design if the concomitant rise in eosinophils is required for the increase in AT remodeling factors or the more fully developed vascular network, thus future studies are needed to address these possibilities.

We observed an increase in AT gene expression of the macrophage marker *Emr1* after initiation of weight loss that resolved by Day 42 of dietary intervention, similar to published work by the Ferrante group (Kosteli et al. 2010); though *Emr1* expression peaked later in our studies than previously reported. This variation in macrophage gene expression may be due to different study designs; our study design relied on a HFD to low‐calorie LFD diet switch with *ad lib* access to food, while the Kosteli et al. (2010) study relied on caloric restriction without switching diets. This may indicate that certain dietary interventions induce varying severities of AT inflammation while resolution occurs. The Kosteli et al. study was published before eosinophils were first discovered in AT (Wu et al. 2011), and thus eosinophils were not measured in that carefully performed study. Our current study therefore complements the existing literature by showing that in addition to macrophages, AT eosinophils also return to comparable levels seen in lean mice during diet‐induced weight loss. We also observed a statistically significant decline in M1‐like proinflammatory macrophage markers *Tnfa* and *Itgax* by the conclusion of diet‐induced weight loss. In contrast, expression of M2‐like anti‐inflammatory macrophage marker, *Arg1*, trended toward an upward surge that paralleled the rise in pan‐macrophage marker *Emr1* before ultimately returning to baseline. Thus, suggesting any increase in total macrophage gene expression at later time points during diet‐induced weight loss was due to anti‐inflammatory resolving Arg1+ macrophages.

Not reported in any study to date, we examined the fluctuation of liver eosinophils across multiple time points during weight loss. Unlike the decline in eosinophils of AT with weight gain, we observe no reduction in liver eosinophils of obese compared to lean mice. Furthermore, while weight loss increased AT eosinophils, liver eosinophils remained constant, despite a reduction in liver weight over time. Interestingly, Kupffer cell (i.e., liver macrophages) numbers initially spiked within 3 days of weight loss but by day 42 liver macrophages dropped below peak Kupffer cell number seen at the height of weight gain; this potentially indicates that liver inflammation recovers quicker than AT inflammation during dietary weight loss.

Our study has shown that during the onset and resolution of obesity, eosinophil populations are regulated in a tissue‐specific manner. Both AT and liver gained significant tissue mass during obesity, largely from extra lipid deposition; however, only in AT did eosinophil levels change during weight gain and weight loss. Furthermore, AT eosinophils correlated with AT remodeling processes during diet‐induced weight loss from an obese state. It is possible eosinophils help orchestrate some of the remodeling processes during obesity as they are known to produce vascular growth factors, collagenase degrading matrix metallopeptidases, and various cytokines and chemokines. While our current study begins exploring the role of eosinophils in AT remodeling during weight loss, there are many more questions and mechanisms for the immunometabolism field to investigate. Though obesity prevention is perhaps the most ideal situation, over one third of persons in the United States, and a growing number worldwide, are already obese and need better treatments to assist in their weight reduction and consequently alleviate the comorbidity risks of obesity. A greater understanding of the mechanisms and cell types involved in returning AT to a lean state offers promise of combating the obesity epidemic by the most effective means possible.

## Conflict of Interest

The authors declare no conflict of interest.
